# Induced DNA hypomethylation by Folic Acid Deprivation in Bovine Fibroblast Donor Cells Improves Reprogramming of Somatic Cell Nuclear Transfer Embryos

**DOI:** 10.1038/s41598-020-61797-3

**Published:** 2020-03-19

**Authors:** Mina Jozi, Farnoosh Jafarpour, Reza Moradi, Faezeh Ghazvini Zadegan, Khadijeh Karbalaie, Mohammad Hossein Nasr-Esfahani

**Affiliations:** 1 Department of Reproductive Biotechnology, Reproductive Biomedicine Research Center, Royan Institute for Biotechnology, ACECR, Isfahan, Iran; 2 ACECR Institute of Higher Education (Isfahan Branch), Isfahan, Iran; 3 Department of Cellular Biotechnology at Cell Science Research Center, Royan Institute for Biotechnology, ACECR, Isfahan, Iran

**Keywords:** Embryology, Epigenetic memory

## Abstract

Aberrant patterns of DNA methylation are consistent events in SCNT derived embryos and mechanistically are believed to be related to abnormal development. While some epigenetic drugs have been used in attempts to improve SCNT efficiency but some concerns remained toward the safety of these drugs on the health of future offspring. Folate is an essential cofactor in one‐carbon cycle for conversion of homocysteine to methionine, thereby ensuring supply of SAM, the universal methyl donor for many biological methylation reactions including DNA methylation. Therefore, *in vitro* DNA hypo-methylation can be induced by folate deprivation and this study aims at deciphering the role of folic acid deprivation in culture medium of BFFs for 6 days on SCNT efficiency. Our data revealed that culture of fibroblast cells in folate− medium containing 0.5% FBS did not alter the cell cycle compared to other groups. Flowcytometric analysis revealed that DNA methylation (5-mC level) in folate deprived cells cultured in 0.5% serum was decreased compared to folate+ group. The result of bisulfite sequencing was in accordance with flowcytometric analysis, which indicated a decrease in DNA methylation of *POU5F1* promoter. Gene expression analysis revealed an increase in expression of *POU5F1* gene in folate− group. The nuclear area of the cells in folate− group was significantly larger than folate+ group. Induced DNA hypomethylation by folate deprivation in the folate− group significantly improved blastocyst rate compared to the folate+ group. DNA methylation level in *POU5F1* promoter and ICR of *H19* and *IGF2* of SCNT derived embryos in the folate− group was similar to the IVF derived blastocysts. In conclusion, our results proposes a promising “non-chemical” instead of “chemical” approach using inhibitors of epigenetic modifier enzymes for improving mammalian SCNT efficiency for agricultural and biomedical purposes.

## Introduction

Somatic cell nuclear transfer (SCNT) technique, as an alternative counterpart for fertilization, comprises erasing epigenetic memory of somatic cell nucleus and establishing new epigenetic information and conferring a totipotent state to the newly forming embryos^1^. The birth of the well-known “Dolly the sheep”, as the result of the first successful SCNT in mammalian species was a metaphor for demonstration of developmental plasticity^2^.

Despite the fact that several mammalian species have been cloned through SCNT technique, the overall efficiency of this technique has remained remarkably low, approximately less than 5% in many species^3^. Generally, SCNT derived embryos suffer from abnormal epigenetic reprogramming, such as DNA/histone hyper-methylation and histone hypo-acetylation. These anomalies hamper early and late embryonic development^4,5^. Based on background literature the most important cause of abnormal epigenetic reprogramming in SCNT embryos is the specific epigenetic characteristic of somatic donor cells, which is very far from the specialized epigenetic status of sperm and oocyte.

One of the most well-known approaches for improving epigenetic reprogramming of reconstructed embryos during SCNT is removal of epigenetic barriers in genome of donor cells and/or reconstructed embryos using epigenetic chemical modifiers or drugs. In addition to this approach, siRNAs targeting of transcripts of epigenetic modifier enzymes remains an alternative approach. Till now, many epigenetic drugs such as DNA methyltransferase inhibitors (DNMTis) and histone deacetylase inhibitors (HDACis) have been used to improve *in vitro* and *in vivo* development of SCNT embryos^6–9^. These two categories of epigenetic modifiers by inducing DNA hypo-methylation and histone hyper-acetylation result in chromatin relaxation and thereby improves nuclear reprogramming.

While some of these epigenetic drugs have remarkably improved the pre- and post-implantation development of SCNT derived embryos^6–9^, but we have some concerns about the side effects of these drugs on the health of future offspring, which remained to be elucidated. Therefore, designing a non-chemical approach which can induce DNA hypo-methylation and/or histone hypo-methylation/hyper-acetylation in donor cells and/or reconstructed embryos is of great interest and importance.

S-adenosyl methionine (SAM) is the predominant methyl donor for many biological methylation reactions including DNA methylation and histone methylation in mammalian cells^10^. In one carbon cycle, remethylation of homocysteine can be carried out via two pathways. In the most common pathway, operating in somatic cells, a methyl group derived from serine, carried by methyl tetrahydrofolate, is transferred to homocysteine by methylenetetrahydrofolate reductase enzyme (MTHFR). In an alternative pathway of methionine production restricted to liver and kidney cells in humans, a methyl group is transferred directly from betaine to homocysteine by betaine-homocysteine methyltransferase (BHMT) enzyme^11,12^. Subsequently, methionine is converted to SAM by addition of adenosine triphosphate by methionine adenosyltransferase^13^.

Researchers have shown that any mutation in MTHFR gene or *in vivo* deficiency of folate leads to DNA hypo-methylation in genomic DNA, which may predispose the individuals to various cancers^14^. Furthermore, *in vitro* folate deprivation result in a significant genomic DNA hypo-methylation in non-transformed cell lines^15^.

Considering that *in vitro* folate deprivation, can induce DNA hypo-methylation this study aims at deciphering the role of folic acid deprivation in culture medium of bovine fibroblast donor cells (BFFs) for 6 days on SCNT efficiency.

## Results

### Bovine fetal fibroblast cells only exhibit expression of MTHFR enzyme

Since in this study we aimed to determine the effect of induced DNA hypo-methylation in fibroblast cells on SCNT efficiency by folate deprivation, mRNA expression of *MTHFR* and *BHMT* mRNA were assessed in both fibroblast and kidney cells to confirm that the only active pathway for methionine production in fibroblast cells is *MTHFR*. In mammalian kidney and liver, homocysteine can also be converted to methionine by two distinct pathways using MTHFR and BHMT enzymes while in other tissues only MTHFR enzyme is responsible for production of methionine.

**Figure 1:**
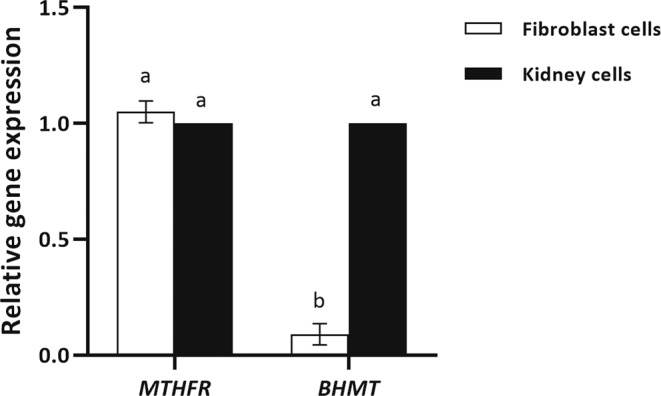
Real-time reverse-transcriptase PCR gene expression analysis of *MTHFR* and *BHMT* in fibroblast cells derived from skin and kidney in bovine. Fold-change values were calculated from triplicate technical replicates of three biological replicates following normalization to *β-ACT*. Values are means ± SEM. Different letters indicate significant differences (*P* < 0.05).

Analysis of relative mRNA expression levels of *MTHFR* and *BHMT* in bovine fibroblast and kidney cells by independent samples t-test revealed a significant lower level of *BHMT* mRNA expression in fibroblast cells relative to *MTHFR* (*P* < 0.05, Fig. 1). Furthermore, gene expression analysis revealed a significant higher expression of *BHMT* in kidney cells versus fibroblast cells (*P* < 0.05, Fig. 1). These data demonstrated that mRNA transcripts for BHMT enzyme is likely to be absent in fibroblast somatic cells in compare to kidney cells and the likely active pathway for methionine production in fibroblast cells appear to be MTHFR.

### Folate deprivation does not reduce fibroblast proliferation

**Figure 2:**
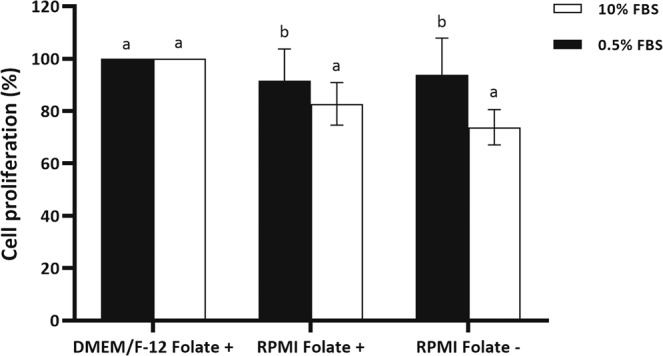
Cell proliferation of BFF cells cultured in DMEM/F-12 Folate+, RPMI Folate+ and RPMI Folate− in presence of 10 (white bar) and 0.5% (black bar) FBS for 6 days. The results were normalized to DMEM/F-12 Folate+ group and are presented as the mean ± SEM. Different letters indicate significant differences (*P* < 0.05).

Previously, several studies have shown that folate deprivation reduces proliferation of various cell types. To determine whether folate deprivation can affect the proliferation of fibroblast cells, the MTS assay was carried out in presence of 10% and 0.5% FBS. Our results revealed that about 6 days of culture in RPMI 1640 medium, both folate sufficient and folate deficient, decreased proliferation rate in compare to DMEM/F-12 folate sufficient medium, routinely used for cell proliferation and starvation in SCNT procedure, in presence of 10% serum (*P* < 0.05, Fig. 2). Furthermore, RPMI 1640 folate deficient medium did not affect the proliferation rate relative to RPMI 1640 folate sufficient medium either in presence of 10% or 0.5% serum (*P* > 0.05, Fig. 2). Finally, neither type of culture medium (DMEM/F-12 or RPMI 1640) nor folate level (folate sufficient medium or folate deficient medium) affected proliferation rate in presence of 0.5% FBS (*P* > 0.05, Fig. 2). All the comparisons were done by one-way ANOVA analysis (α = 0.05) followed by Tukey HSD test.

### Folate deprivation induces arrest in S-phase in fibroblast cells

**Figure 3:**
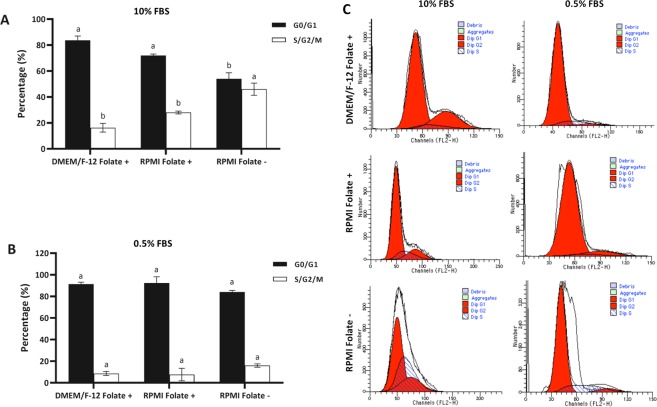
Cell cycle distribution of BFF cells propagated in DMEM/F-12 Folate+, RPMI Folate+ and RPMI Folate− in presence of (**A**) 10 and (**B**) 0.5% FBS growth medium for 6 days. (**C**) Flow cytometry histograms indicating the cell cycle distribution of above mentioned BFF cells. Different letters indicate significant differences (P < 0.05).

To determine the effect of folate deficiency on cell cycle of BFF cells, cell cycle analysis was performed after 6 days of culture. It was shown that cells lacking folate accumulate in the S phase possibly due to nucleotide imbalance and slow DNA synthesis. As depicted in Fig. 3A,C, analysis of cell cycle of cells cultured in DMEM/F-12 folate sufficient medium by one-way ANOVA followed by Tukey HSD test revealed a cell cycle distribution similar to that of cells cultured in RPMI 1640 folate sufficient medium in presence of 10% FBS (*P* > 0.05), whereas cells cultured in RPMI 1640 folate deficient medium showed a significant increase in the proportion of cells in the S phase in presence of 10% FBS (*P* < 0.05, Fig. 3), suggesting that folate-deprived cells have an impaired capacity to synthesize DNA and to complete the cell cycle. Furthermore, in presence of 0.5% serum, presence or absence of folate in culture medium did not affect the distribution of various stages in cell cycle (*P* > 0.05, Fig. 3B,C).

### Folate deprivation does not increase DNA damage in fibroblast cells

**Figure 4:**
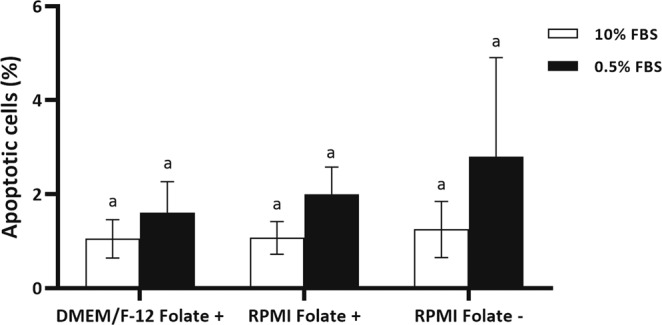
Analysis of apoptotic cells by TUNEL staining of BFF cells propagated in DMEM/F-12 Folate+, RPMI Folate+ and RPMI Folate− in presence of 10 (white bar) and 0.5% (black bar) FBS for 6 days. Values represent the mean ± SEM number of TUNEL-positive cells. Different letters indicate significant differences (P < 0.05).

Folate deprivation has been linked to increased DNA breaks owing to excessive uracil incorporation. DNA double-strand breaks is the most damaging DNA breaks in terms of genome integrity. In this study, terminal deoxynucleotidyl transferase-mediated dUTP nick-end labelling (TUNEL) immunostaining was carried out to detect cellular DNA fragmentation and to assess apoptosis in fibroblast cells cultured in folate deficient culture medium. Analysis of apoptotic cells by one-way ANOVA followed by Tukey HSD test revealed no significant elevation in TUNEL positive cells in presence or absence of folate in culture medium (DMEM/F-12 Folate+ vs. RPMI Folate+ vs. RPMI Folate−) in presence of 10% and 0.5% serum (*P* > 0.05, Fig. 4).

### Effect of folate deprivation on global DNA methylation

The impact of folate deprivation on global DNA methylation in BFF cells was investigated using quantitative flow cytometry. BFF cells treated with folate sufficient and deficient culture medium in presence of 10% and 0.5% FBS for 6 days were used for detection of global DNA methylation.

**Figure 5:**
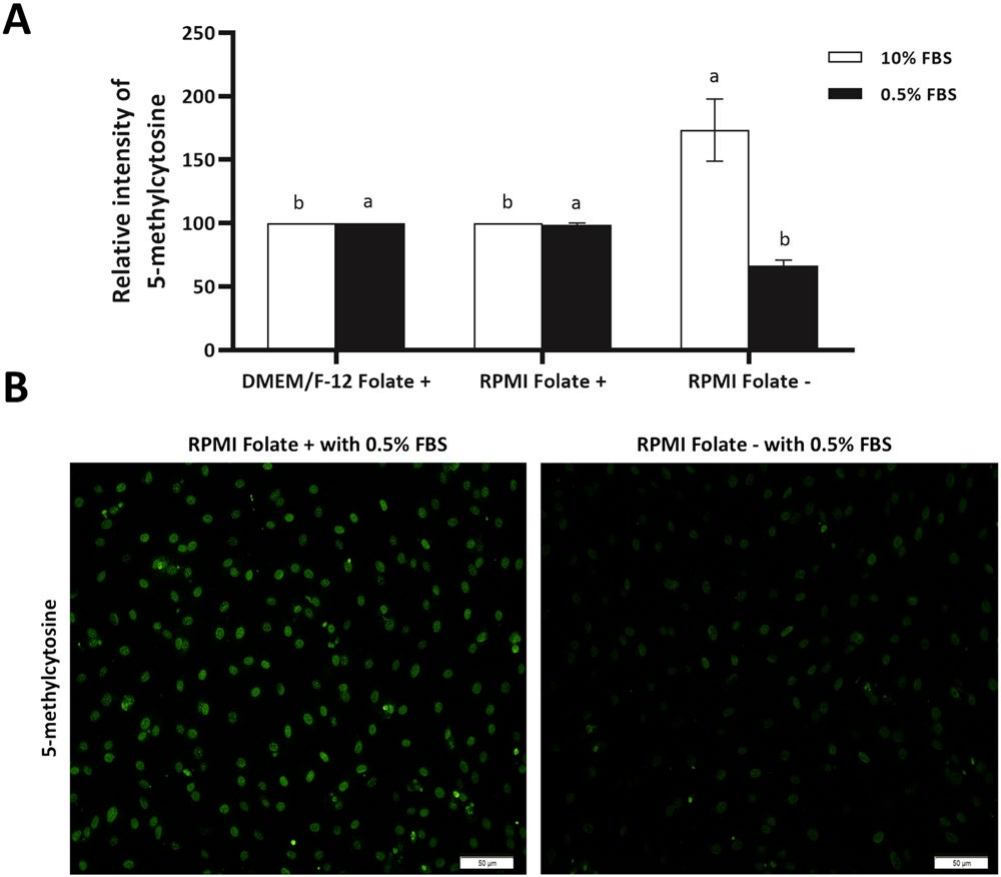
(**A**) Flowcytometry assay for relative level of DNA methylation was performed on BFF cells propagated in DMEM/F-12 Folate+, RPMI Folate+ and RPMI Folate− in presence of 10 (white bar) and 0.5% (black bar) FBS for 6 days. Values represent the mean ± SEM. Different letters indicate significant differences (P < 0.05). (**B**) Immunostaining of BFF cells propagated in RPMI Folate+ and RPMI Folate− in presence of 0.5% FBS for 5-mC. Scale bars represent 50 µm.

The flow cytometry data analysed by one-way ANOVA followed by Tukey HSD test suggested a significantly lower degree of genomic DNA methylation in the folate deprived cells, compared with the folate-sufficient cells in presence of 0.5% serum (*P* < 0.05, Fig. 5A,B). Interestingly, we observed higher degree of genomic DNA methylation in the folate deprived cells, compared with the folate sufficient cells in presence of 10% serum (*P* < 0.05, Fig. 5A). The mechanism of this phenomenon is unknown and needs to be studied in detail.

### Effect of folate deprivation on H3K9me2

**Figure 6:**
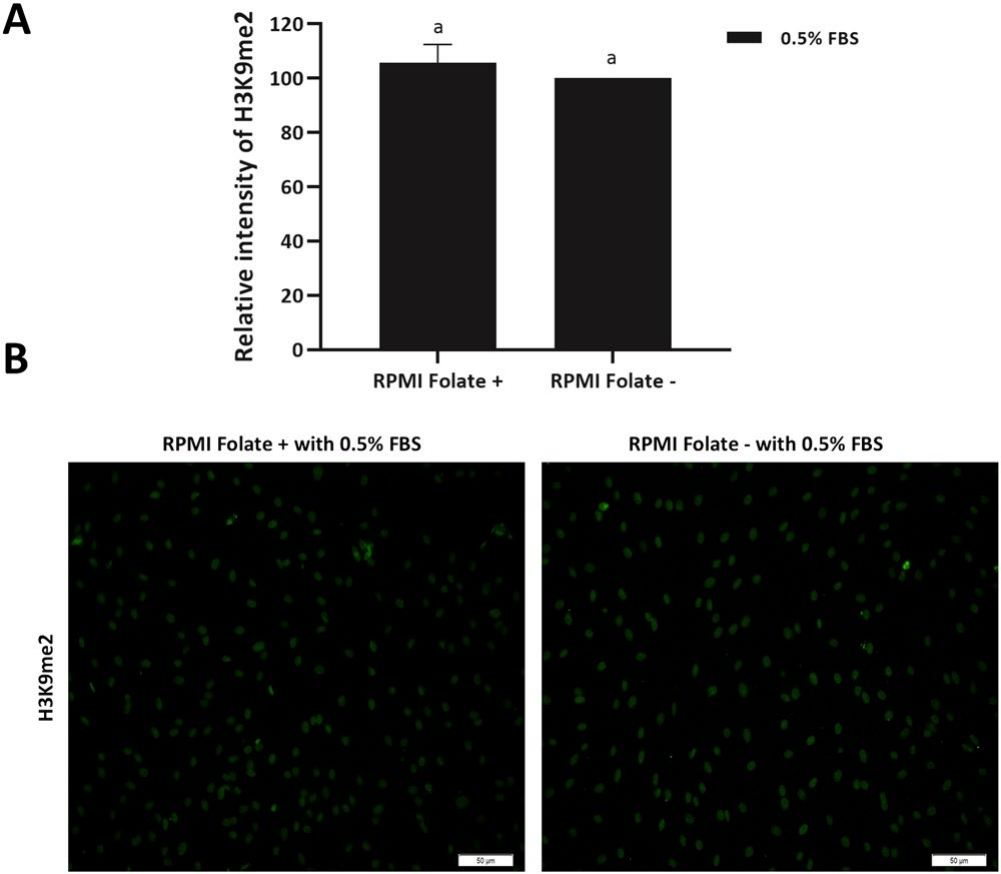
(**A**) Flowcytometry assay for relative H3K9me2 was performed on BFF cells propagated in RPMI Folate+ and RPMI Folate− in presence of 0.5% FBS for 6 days. Values represent the mean ± SEM. Different letters indicate significant differences (P < 0.05). (**B**) Immunostaining of BFF cells propagated in RPMI Folate+ and RPMI Folate− in presence of 0.5% FBS for H3K9me2. Scale bars represent 50 µm.

The impact of folate deprivation on H3K9me2 in BFF cells was investigated using quantitative flow cytometry and data analysed by independent samples t-test. Interestingly, we observed that the value for H3K9me2 remained unchanged following 6 days folate deprivation in culture medium in presence of 0.5% FBS (RPMI Folate+ vs. RPMI Folate−) (*P* > 0.05, Fig. 6A). In addition, immunocytochemistry of BFF cells revealed that intensity of H3K9me2 in RPMI Folate+ group is similar to RPMI Folate− group (Fig. 6B).

### Fibroblast cells in folate deficient medium demonstrate larger nuclear area than their counterparts in folate sufficient medium

**Figure 7:**
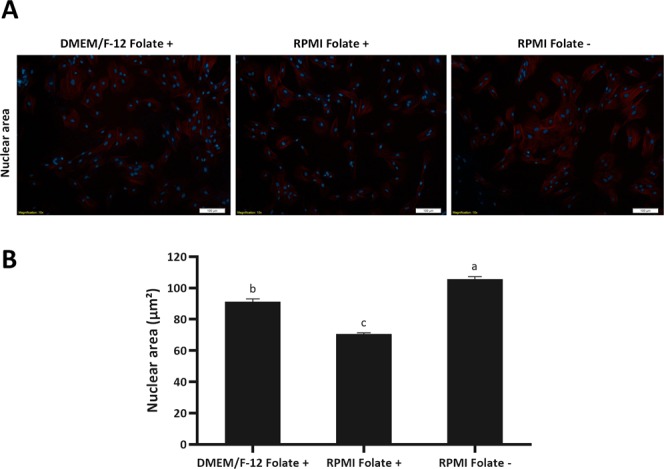
(**A**) The nuclear area of folate deprived cells was assessed by fluorescence microscopy after phalloidin-tetramethylrhodamine B isothiocyanate and Hoechst 33342 staining. Merged images of tubulin and DNA are shown. Scale bars represent 100 µm. (**B**) Using ImageJ software (National Institute of Mental Health, Bethesda, Maryland, USA) the nuclear area of 100 fibroblasts in 10 different fields was calculated. Values represent the mean ± SEM. Different letters indicate significant differences (P < 0.05).

We compared mean nuclear area in fibroblast cells serum starved in folate deficient medium against their counterparts in folate sufficient medium. By staining cells with Hoechst 33342 and measuring the nuclear area with ImageJ software and analysis by one-way ANOVA followed by Tukey HSD test, we found that mean nuclear area in fibroblast cells in folate deficient medium is significantly higher than fibroblast cells in folate sufficient medium (DMEM/F-12 and RPMI 1640 medium) (*P* < 0.05, Fig. 7A,B), suggesting a more relaxed chromatin configuration in folate deficient culture medium. Furthermore, our data revealed a significant smaller mean nuclear area in RPMI 1640 folate sufficient medium against to fibroblast cells in DMEM/F-12 folate sufficient culture medium (*P* < 0.05, Fig. 6), indicating a more compact chromatin state of fibroblast cells in RPMI 1640 medium in compare to DMEM/F-12 medium.

### Fibroblast cells in folate deficient medium demonstrate lower genomic DNA methylation in promoter of POU5F1 gene but not in ICR of H19/IGF2 gene and promote expression of POU5F1

**Figure 8:**
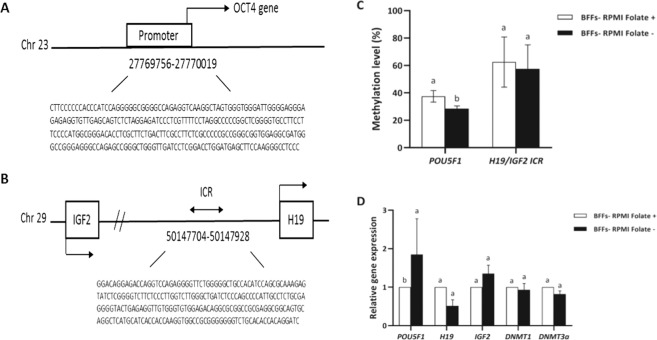
Schematic representation of the bovine (**A**) *H19/IGF2* ICR region and (**B**) *POU5F1* promoter with gene sequences used for methylation sequencing. (**C**) Quantitative analysis of 5mC levels in promoter of *POU5F1* gene and ICR of *H19/IGF2* imprinted genes in BFF cells propagated in RPMI Folate+ and RPMI Folate− culture medium in presence of 0.5% serum for 6 days. (**D**) RT-qPCR analysis of *POU5F1*, *H19*, *IGF2*, *DNMT1* and *DNMT3A* expression in BFF cells propagated in RPMI Folate+ and RPMI Folate− culture medium in presence of 0.5% serum for 6 days. Values represent the mean ± SEM. Different letters indicate significant differences (P < 0.05).

Following the initial characterization of global DNA methylation in fibroblast cells in folate deficient culture medium by flow cytometry, we examined DNA methylation level of ICR of *H19/IGF2* (an imprinting gene) (Fig. 8A) and *POU5F1* promoter (a non-imprinting gene) (Fig. 8B) using bisulfite sequencing analysis and data were analysed by independent samples t-test. In addition, mRNA expression of assessed genes was analysed by independent samples t-test. Meanwhile, culture of fibroblast cells in folate deficient medium for 6 days significantly reduced DNA methylation level of *POU5F1* promoter (*P* < 0.05, Fig. 8C), consequently leading to increased expression levels of *POU5F1* (*P* < 0.05, Fig. 8D). However, no differences in DNA methylation level (*P* > 0.05, Fig. 8C) and gene expression of *H19* and *IGF2* imprinted genes (*P* > 0.05, Fig. 8D) were observed between the fibroblast cells in folate deficient medium and their counterparts in folate sufficient medium. All together, these results suggested that folate deficient medium can initiate DNA demethylation and promote expression of *POU5F1* in bovine fibroblast cells. In addition, no changes were observed in the expression levels of *DNMT3A* and *DNMT3B* between the fibroblast cells cultured in folate sufficient and deficient medium (*P* > 0.05, Fig. 8D).

### Reconstructed oocytes by fibroblast cells cultured in folate deficient medium exhibited higher developmental potential than those cultured in folate sufficient medium

Using BFF cells treated for 6 days in folate deficient culture medium (RPMI 1640 Folate −) as somatic donor cells, we generated SCNT embryos and examined their preimplantation development in compare to those generated by fibroblast cells cultured for 6 days in folate sufficient medium (DMEM/F-12 Folate+ and RPMI 1640 Folate+). Analysis of cleavage and blastocyst rates by one-way ANOVA followed by Tukey HSD test revealed that while there was no difference between the cleavage rate of DMEM/F-12 Folate+, RPMI 1640 Folate+ and RPMI 1640 Folate− groups but the RPMI 1640 Folate− group demonstrated significant higher blastocyst yield (26.56 ± 2.90) RPMI 1640 Folate+ (13.83 ± 3.64) group (*P* < 0.05, Table 1). Blastocyst yield in RPMI 1640 Folate− group, however, was not significantly higher than DMEM/F-12 Folate+ group (21.58 ± 3.91) (*P* > 0.05, Table 1).

**Table 1:** Effect of folate deprivation on development competence of the SCNT derived embryos.

Groups	No. of replicates	No. of embryos cultured	Embryo development	
			No. of Cleaved embryos (%)	No. of blastocysts on day 7 (%)
DMEM/F-12 Folate+	5	128	101 (85.97 ± 5.08)^a^	18 (21.58 ± 3.91)^ab^
RPMI Folate+	7	167	128 (81.54 ± 2.76)^a^	19 (13.83 ± 3.64)^b^
RPMI Folate−	9	253	208 (87.66 ± 1.79)^a^	52 (26.56 ± 2.90)^a^

### Treating of fibroblast cells with folate deficient culture medium can partially rectify the hyper-methylation state of POU5F1 promoter gene and promote its expression in SCNT embryos but not in H19/IGF2 gene

**Figure 9:**
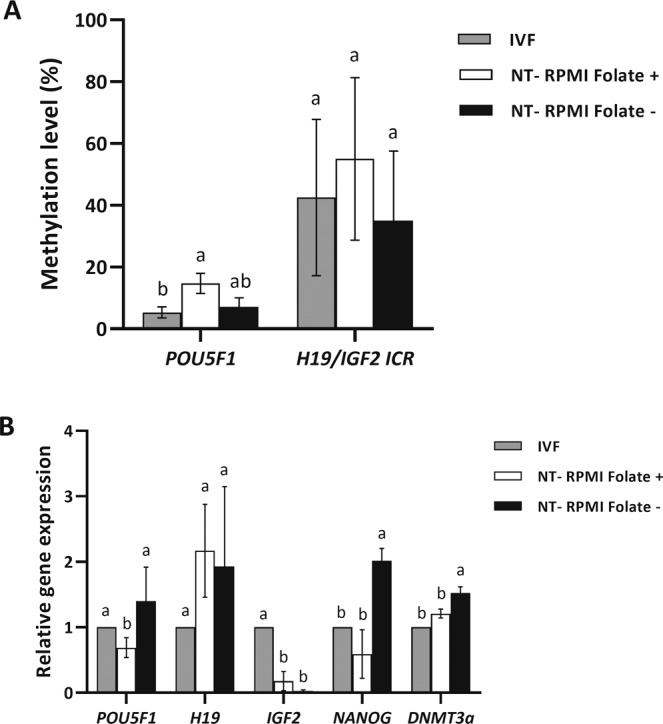
(**A**) Quantitative analysis of 5mC levels promoter of *POU5F1* gene and ICR of *H19/IGF2* imprinted genes in blastocyst embryos in IVF, NT-RPMI Folate+, and NT-RPMI Folate− groups. (**B**) RT-qPCR analysis of *POU5F1*, *H19*, *IGF2*, *NANOG* and *DNMT3A* expression in IVF, NT-RPMI Folate+, and NT-RPMI Folate at blastocyst stage. Values represent the mean ± SEM. Different letters indicate significant differences (P < 0.05).

We also detected DNA methylation level of *POU5F1* promoter and ICR of *H19/IGF2* imprinted gene in blastocyst embryos of IVF, SCNT-RPMI Folate+, and SCNT-RPMI Folate− groups by bisulfite sequencing assay and data were analysed by one-way ANOVA followed by Tukey HSD test. The DNA methylation level of *POU5F1* promoter in SCNT-RPMI Folate+ was significantly higher than IVF and SCNT-RPMI Folate− (*P* < 0.05, Fig. 9A), whereas this level in SCNT-RPMI Folate− group was similar to IVF group (*P* > 0.05, Fig. 9A). The DNA methylation level of H19/IGF2 ICR was similar between IVF, SCNT-RPMI Folate+ and SCNT-RPMI Folate− groups (*P* < 0.05, Fig. 9A).

To further confirm the regulatory relationship between folate deprivation and embryonic development, we performed RT-qPCR to detect expressions of key pluripotency genes (*POU5F1* and *NANOG*) and imprinted genes (*H19* and *IGF2*) in blastocysts of IVF, SCNT-RPMI Folate+, and SCNT-RPMI Folate− groups. Analysis of the assessed genes by one- way ANOVA followed by Tukey HSD test revealed significantly lower level of expression of *POU5F1* and *NANOG* in the SCNT- RPMI Folate+ group relative to that of IVF blastocysts and SCNT-RPMI Folate− group (*P* < 0.05, Fig. 9B). However, SCNT- RPMI Folate− blastocysts showed *POU5F1* and *NANOG* mRNA level approximating that of the IVF group (*P* > 0.05, Fig. 9B). We also noted that *H19* mRNA level did not show significant difference among the three aforementioned groups (*P* > 0.05, Fig. 9B).

These findings suggested that treatment of donor fibroblast cells with folate deficient culture medium can promote *POU5F1* and NANOG expression in SCNT embryos.

## Discussion

In this study, we developed a non-chemical approach by altering DNA methylation of fibroblast donor cells by deprivation of folic acid in cell culture medium, and we acknowledged that deprivation of folic acid for 6 days in presence of 0.5% FBS improves developmental competence of bovine SCNT embryos.

In order to make sure that the only mechanism for producing methionine in fibroblast cells is MTHFR enzyme, and folic acid deprivation can efficiently induce DNA hypo-methylation in fibroblast cells, we assessed the expression of MTHFR and BHMT enzymes in fibroblast cells. In consistent with previous studies^11,16^, our data revealed that BHMT enzyme is likely to be absent or very low in fibroblast somatic cells and the only active pathway for methionine production in fibroblast cells appear to be MTHFR.

In examining the effect of folic acid deprivation on SCNT efficiency, the type of culture medium used is very important. DMEM/F-12 is a common culture medium for culturing fibroblast cells but unfortunately, DMEM/F-12 folic acid free medium is not available. Therefore, we focused on RPMI 1640 medium with and without folic acid for assessing the effect of folic acid deprivation on SCNT efficiency. Considering that DMEM/F-12 is a common culture medium for culturing fibroblast cells, thereby, we used all three culture medium (DMEM/F-12 Folate+, RPMI 1640 Folate+ and RPMI 1640 Folate−) throughout our study.

The effect of folic acid free culture medium (RPMI 1640 Folate−) for 6 days in presence of 10% serum surprisingly and contrary to our expectations, revealed that the level of DNA methylation in RPMI 1640 Folate− medium is significantly higher than DMEM/F-12 and RPMI 1640 Folate+ medium. One explanation for this observation is that the available amount of folic acid in FBS is relatively lower than the amount present in DMEM/F-12 and RPMI 1640 Folate+ medium (6.0 µM vs. 2.3 µM) and cells through compensatory mechanism may uptake more folic acid and have a more active one carbon metabolism cycle in compare to other groups, which needs more investigations to be elucidated.

Increasing evidence indicates that folate plays important roles in protein synthesis, cell division and growth. In addition folate is a methyl donor that plays a critical role in methylation reactions, including DNA methylation. Many *in vivo* studies and animal models demonstrated the relation between folate deficiency and the development of genomic DNA hypomethylation, which is an early epigenetic found in many cancers including colorectal, gastric and breast cancers.

There are few and sparse data about the folate deprivation in *in vitro* studies. Based on the literature, supplementation of reduced folate media with FBS can maintain the normal growth of cultured cells because FBS contains folate in an amount which is sufficient for cell survival^17^. Regard to this, Stempak and colleagues used dialyzed serum in order to eliminate folic acid from the serum for assessing the effect of folate deprivation on methionine cycle intermediates and DNA methylation^15^. Interestingly, when we reduced the serum in culture medium from 10% to 0.5%, we observed that cells cultured in RPMI 1640 folic free medium (RPMI 1640 Folate−) present lower global DNA methylation level in compare to other groups. As SAM is the universal methyl donor for histone methylation and abnormal histone methylation has also been considered as a barrier to the reprogramming of bovine SCNT embryos, we also investigated the effects of folate deprivation in fibroblast donor cells on H3K9me2 of fibroblast cells. Interestingly, we observed that the value for H3K9me2 remained unchanged following 6 days folate deprivation in culture medium in presence of 0.5% FBS. Studies on folate deprivation impact on histone methylation are sparse. In liver samples of mouse fed with a folate-deficient diet, H3K4me was increased but the value for H3K9me2 remained unchanged^18^. A knockdown of MTHFR in HeLa cells causes a decrease in H3K9me3 and an increase in centromeric satellite repeat expression^19^. Upon choline deprivation of mouse neural-progenitor cells, H3K9me2 is modified at specific genomic locus, causing poor recruitment of G9a^20^. The fact that value for H3K9me2 is unchanged by folate deficiency in the current study, unlike the differences observed for DNA methylation, however, remained to be elucidated.

To further investigate the effect of folic insufficiency on site-specific DNA methylation of cells propagated in absence of folate, we assessed the DNA methylation of *POU5F1* promoter as a non-imprinting gene and ICR of *H19*/*IGF2* as an imprinting gene. While we observed a DNA hypo-methylation in *POU5F1* gene, no changes were observed in ICR of *H19*/*IGF2*. These results possibly indicate that imprinted genes are recalcitrant to epigenetic changes which is consistent with the role envisaged for imprinted genes^21^. Furthermore, these result suggest that promoter of genes related to pluripotency are prone to relaxation by this approach^22,23^.

Several studies have shown that folate deprivation reduces proliferation of various cell types^24,25^. Furthermore, it has been shown that cells lacking folate accumulate in the S phase^26^ and present increased uracil mis-incorporation which eventually end in DNA damage. In accordance with previous studies, we also observed that higher percentage of cells are located in S phase in RPMI Folate− in compare to other groups which is likely related to nucleotide imbalance and slow DNA synthesis in folate deprived cells in compare to cells supplement with adequate amount of folate.

In contrary, we observed that neither type of culture medium (DMEM/F-12 or RPMI 1640) nor folate deprivation affected DNA damage in presence of 10 or 0.5% FBS. These insignificant changes may be result from short *in vitro* culture period and extended exposure to this condition may result in lower proliferation rate and higher DNA damage.

By assessing the effect of folic acid deprivation in RPMI 1640 medium on efficiency of SCNT, we observed a two fold increase in blastocyst rate which is in accordance with the characteristic of folate deprived cells such as reduced global and site-specific DNA methylation and increased nuclear area related to relaxation of chromatin. These observations indicate that RPMI Folate− culture medium induces a more favorable condition for better epigenetic and chromatin make up for SCNT procedure. It was also interesting to note that, while there was no difference in the various cellular characteristics of DMEM/F-12 Folate+ and RPMI 1640 Folate+ medium, no difference was also observed in the efficiency of SCNT between the two medium.

Our data revealed that the epigenetic signature of *POU5F1* promoter of blastocysts in RPMI Folate− group is similar to IVF group. To further verify the beneficial effect of *in vitro* folate deprivation, we compared the relative expression of some developmentally important genes between IVF, RPMI Folate+ and RPMI Folate− groups. Gene expression analysis illustrates that the relative expression of *NANOG* as a gene related to developmental competency and DNMT3 required for establishing DNA methylation after DNA demethylation during preimplantation stage was higher in blastocysts from RPMI Folate− compared with RPMI 1640 Folate+ group and was similar to IVF derived blastocysts, indicating the beneficial effect of global DNA hypo-methylation induced by folate deprivation on blastocyst quality.

## Conclusion

In summary, we have established an improved SCNT method by inducing a folate deprivation *in vitro*. Using this method, we observed a two fold increase in blastocyst rate in RPMI 1640 Folate− group in compare to RPMI 1640 Folate+ group and subsequently the derived blastocysts have similar gene expression and epigenetic signature to IVF blastocysts than those in RPMI 1640 Folate+ group. The results of this study may provide a promising non-chemical approach instead of using chemical inhibitors of epigenetic modifier enzymes for improving mammalian cloning efficiency for agricultural and biomedical purposes.

## Materials and methods

### Media and reagents

Unless stated differently, all chemicals and media were obtained from Sigma Aldrich Chemical Co. (St. Louis, MO) and Gibco (Grand Island, NY), respectively. The Institutional Review Board and Institutional Ethical Committee of the Royan Institute approved all animal care protocols and the proposal. In addition, all methods were performed in accordance with the Institutional Review Board and Institutional Ethical Committee of the Royan Institute guidelines and regulations.

### Experimental design

At passage 3–4, fibroblast cells were added in equal densities to 6-mm culture dishes containing DMEM/F12 (Gibco 32500–035, USA), RPMI 1640 Folate+ (Biowest L0498, France) and RPMI 1640 Folate− (Gibco 27016–021, USA) plus 10% or 0.5% FBS (Gibco 10270, USA). In presence of 10% FBS cells were passaged every 3 days and cultured for 6 days. For treatment of donor cells in culture medium containing 0.5% FBS, primarily they were cultured in presence of 10% FBS for 3 days and subsequently the amount of FBS was reduced to 0.5% FBS for 6 days. At the end of treatment, cells were used for analyzing of different cellular characteristics such as cell proliferation, cell cycle, DNA fragmentation, nuclear area, evaluation of DNA methylation and H3K9me2 using immunostaining with flow cytometry, evaluation of DNA methylation of promoter of *POU5F1* gene and ICR of *H19/IGF2* imprinted genes using bisulphite sequencing and gene expression as described below. Finally treated cells were used for SCNT. All the experiments were carried out in triplicates.

### Bovine fetal fibroblast cells (BFFs) collection

BFFs were isolated from an approximately 2-month-old female fetus. Briefly, after removal of head, limbs and viscera, remaining tissue was washed in phosphate buffered saline without calcium and magnesium (PBS^−^) (Gibco 21600, USA). The tissue was finely minced using a sterile razor blade until it becomes possible to pipette. Subsequently, the minced tissue was dissociated with 0.25% trypsin/EDTA (Gibco 25300, USA) for 10 min at 37 °C. The cell suspension was washed, centrifuged and cultured in Dulbecco’s modified Eagle medium F-12 (DMEM/F-12) containing 10% FBS and 1% penicillin-streptomycin (Pen/Strep) (Gibco 15140122, USA) at 37.5 °C and 5% CO_2_ in a humidified atmosphere. After reaching confluency, BFFs were harvested and frozen. Frozen stocks were thawed and fibroblast at passage 3–4 were used for various experiments.

### Cell culture of BFF donor cells

At passage 3–4, BFFs were cultured in DMEM/F-12 containing 6 µM folic acid (DMEM/F-12 Folate+, control group), RPMI 1640 medium containing 2.3 µM folic acid (RPMI 1640 Folate+, RMPI 1640 control group) and folic acid free RPMI 1640 medium (0 mM folic acid) (RPMI 1640 Folate−, deprived group), which were supplemented with 10% or 0.5% FBS and 1% Pen/Strep. Cells were passaged every 3 days and cultured for 6 days in presence of 10% FBS. For treatment of donor cells in culture medium containing 0.5% FBS, primarily they were cultured in presence of 10% FBS for 3 days and subsequently the amount of FBS was reduced to 0.5% FBS for 6 days.

### Cell proliferation assessment

Proliferation of fibroblast cells in presence or absence of folic acid was determined using 3- (4, 5-dimethylthiazol-2-yl)-5-(3-carboxymethoxyphenyl)-2-(4-sulfophenyl)-2H-tetrazolium (MTS) assay (Promega G3582, USA). In brief, 1000 and 15000 BFF cells were cultured in presence of 10% and 0.5% FBS in DMEM/F-12 Folate+, RPMI 1640 Folate+ and RPMI 1640 Folate− culture medium in 96-well dish. After the desired times, MTS was added to each well and incubated for 4 h at 37 °C. Absorbance ratio of cultured cells in RPMI 1640 Folate+ and RPMI 1640 Folate− relative to DMEM/F-12 Folate+ was measured at 492 nm by using multi-well spectrophotometer. All analyses were measured in three independent replicates and each replicate consisted of triplicate samples.

### Cell cycle assessment

Cell cycle analysis was carried out by flow cytometry as described by Jafarpour *et al*.^27^. Briefly; cultured cells in different culture medium were trypsinized and resuspended in ice-cold 75% ethanol for 1 h. The fixed cells were resuspended in staining solution containing propidium iodide (50 µg/mL), RNase A (100 µg/mL), and Triton X-100 (0.5%) for 20 min in the dark at room temperature (RT). In order to remove the aggregated cells, the cell suspension was filtered through 40 µm nylon mesh. Finally, a total of 20,000 stained cells were collected on a fluorescence-activated cell sorter (FACS) Caliber (Becton Dickinson, San Jose, CA) and were analyzed using Modfit software^28^.

### DNA fragmentation analysis

To assess the effect of folate deprivation on DNA fragmentation, treated cells were stained using terminal deoxynucleotidyl transferase (TdT)-mediated dUTP-digoxigenin nick end labeling (TUNEL) with an *in situ* cell-death detection kit (Promega_ Diagnostic Corporation, Madison, WI, USA). Briefly, according to the manufacturer’s guidelines, cells were fixed with paraformaldehyde for 20 min at 4 °C. Subsequently, cells were permeabilized with 0.2% Triton X-100 for 15 min at RT. After removing the permeabilization solution, cells were incubated with equilibration buffer (EB) for 5 min at RT. Then cells were resuspended in 50 μl of rTdT incubation buffer (45 μl EB + 1 μl rTdT enzyme + 5 μl nucleotide mix) and incubated in a water bath for 60 minutes at 37 °C in the dark. The reaction was stopped with 1 ml of 20 mM EDTA. After that, cells were washed with PBS^−^ containing 0.1% Triton X-100 ® and 5 mg/ml BSA. Finally, cells were analyzed with a FACS- Calibur™ flow cytometer (Becton Dickinson, San Jose, CA).

### Nuclear area assessment

To assess the effect of presence or absence of folic acid on chromatin structure, fibroblast cells in various treatment groups were fixed and stained with Hoechst 33342. Nuclear area of 100 fibroblasts in 10 different fields was calculated using ImageJ software (National Institute of Mental Health, Bethesda, Maryland, USA).

### Assessment of epigenetic marks in fibroblasts, 5-methylcytosine and H3K9me2

#### Flow cytometry

The effect of folic acid deprivation on global DNA methylation and H3K9me2 of fibroblast cells were assessed using immunostaining with flow cytometry through measuring the fluorescence intensity of the complexes between primary and secondary antibodies in the cells as described in our previous papers^29^. In brief, after trypsinization, cells were fixed with 70% ethanol for 1 h in 4 °C. Permeabilization was carried out using 1% Triton X-100 in PBS- for 30 min at RT. For detection of 5-methylcytosine DNA was denatured with HCl (4 N) and then the effect of HCl was neutralized with Tris-HCL (0.1 M). Subsequently, after blocking the non-specific binding site with blocking solution for 2 h at RT, cells were incubated with primary antibody (5-mc, Eurogentec BI-MECY-0500, Belgium or H3K9me2, Abcam ab1220) for overnight in 4 °C. After extensive washing, cells were incubated with secondary antibody (Chemicon AP124F) for 1 h at 37 °C. Cells were collected with the FACS-Caliber and were analyzed using CELL QUEST 3.1 software (Becton Dickinson). Three replicates were conducted for each treatment with appropriate controls to eliminate the possible effects of auto-fluorescence and nonspecific binding by the secondary antibody.

#### Analysis of DNA methylation using bisulfite sequencing PCR

Bisulfite sequencing was carried out based on previous studies^30^. In brief, genomic DNA was extracted using DNeasy® Blood and Tissue Kit (Qiagen 69504). Then DNA was treated for bisulfite sequencing according to the user manual of EpiTect® Bisulfite Kit (Qiagen, Germany).

Meth Primer online software was chosen for primer design (www.urogene.org/MethPrimer) (Table 2). The PCR master mix include 300 ng of each forward and reverse primers, 4 μl bisulfite modified DNA, 1 × ammonium sulfate (AMS) buffer (Cinna Gen, Iran), 6.7 mM MgCl_2_ (Cinna Gen, Iran), 1.25 mM dNTP (Cinna Gen, Iran), 0.6 USmar Taq (Cinna Gen) and 10 mM 2-mercaptoethanol (Sigma) in a 50 μl reaction volume. PCR reaction was set based on following program: 95 °C for 10 min, 39 cycles at 95 °C for 30 sec, 63 °C for 30 sec, and 72 °C for 1 min, followed by a final extension at 72 °C for 10 min^31^. Afterward, PCR products were subcloned into a pTZ57R/T cloning vector (InsTAcloneTM PCR Cloning Kit, Fermentas) as described in the manufacturer’s protocol. Ligated vectors were transferred into the DH5α strain of E. coli and at least 10 positive colonies, which were selected by PCR analysis through M13 forward and reverse primers, were extracted by Qiaprep® Spin Miniprep Kit (Qiagen) and sequenced. The sequence of primers is listed in Table 2. The sequences were analyzed with bisulfite sequencing DNA methylation analysis (BISMA) online software^32^.

**Table 2:** Primers used in this study for bisulfite sequencing analysis.

Gene	Forward primer (5′-3′)	Reverse primer (5′-3′)	AT*	Product size
*POU5F1*	GTTAGAGGTTAAGGTTAGTGGGTG	AAAAACCCTTAAAAACTCATCCAA	63 °C	235 bp
*H19/IGF2* ICR	GGATAGGAGATTAGGTTTAGAGGG	AATCCTATAATATACAAACCCCC	66 °C	225 bp

### RNA extraction and gene expression analysis in fibroblast cells

Total RNA from fibroblast cells was extracted by RNeasy Mini Kit (QIAGEN 74106) according to manufacturer’s instruction. cDNA synthesis was performed with 1 μg of total RNA implementing random hexamer primer and RevertAid™H First Strand cDNA Synthesis Kit (Takara RR037A). Real time PCR was performed with SYBR Green PCR Master Mix (TaKaRa) in a Thermal Cycler Rotor-Gene 6000 (Corbett, Mortlake, Australia), according to the manufacturer’s protocol. Expressions of target genes were normalized to *B-actin* gene expression level. All measurements were carried out in triplicate, from three separate samples, and data were analyzed using the 2^−ddCt^ method. The sequence of primers is listed in Table 3.

**Table 3:** List of primers used in this study for real time PCR.

Gene	Forward primer (5′-3′)	Reverse primer (5′-3′)	AT*	Product size	Accession number
*B-ACTIN*	TTCCTGGGTATGGATCCTG	GGTGATCTCCTTCTGCATCC	58 °C	130 bp	XM_015467124.1
*MTHFR*	AAGATGAAGCGGAAGATG	AACCTGGAGATGAGATTG	48 °C	98 bp	NM_001011685.1
*BHMT*	CAGACCTTCACCTTCTATGC	CTCCTTCATCAGCCACTTG	56 °C	130 bp	NM_001011679.1
*POU5F1*	GGAAAGGTGTTCAGCCA	ATTCTCGTTGTTGTCAGC	62 °C	123 bp	NM_174580.3
*NANOG*	TTGTGACGGCTATTGTATG	ACCTCTTACTGGACTCATT	53 °C	159 bp	NM_001025344.1
*DNMT3a*	TGGTCCTGGGCGTTAG	CCTGCTTTATGGAGTTCG	57 °C	252 bp	NM_001206502.1
*H19*	TCAGCCCCGAGACCAC	ACGCTCAGAGACCAGG	53 °C	327 bp	XM_001256398.4
*IGF2*	CCTGCTGGAGACTTACTG	CTTGGCGAGCGTGCGA	62 °C	199 bp	XM_015461332.1

### Preparation of bovine ovaries and oocyte collection

Ovaries were obtained from cows at local slaughterhouse (Fasaran, Isfahan), with the permission of the manager of the slaughterhouse and the agreement of veterinary organization. Ovaries were collected from slaughterhouse at 2–4 p.m. and transported to the laboratory at 15–17 °C by 6 p.m. Immediately, after receiving the ovaries, they were washed, trimmed and stored at 15 °C until time for harvesting the oocytes based on previously set protocols^33,34^. The cumulus oocyte complexes (COCs) were aspirated from 2–8 mm follicles using an 18 gauge needle attached to a vacuum pump. Only oocytes possessing homogenous cytoplasm and at least three layers of compact cumulus cells were selected for *in vitro* maturation (IVM).

### IVM of bovine oocyte

The procedure of IVM of oocytes was according to Hosseini *et al*.^35^. In brief, selected COCs were primarily washed in hepes-tissue culture medium 199 (H-TCM) and subsequently in TCM medium supplemented with sodium pyruvate, 10% FBS, 10 µg/ml follicle stimulating hormone (FSH; Sigma F8174), 10 µg/ml luteinizing hormone (LH; Sigma L5269), 100 mM 17 β-estradiol (E2; Sigma E4389), 0.1 mM cysteamine (Sigma M9768), 10 ng/ml epidermal growth factor (EGF; Sigma E4127) and 100 ng/ml insulin-like growth factor 1 (IGF1; R&D 291-G1) (maturation medium). COCs were then transferred and cultured in groups of 10 into 50 μl droplets of maturation medium and incubated for 22–24 h at 38.5 °C in a humidified 5% CO_2_ atmosphere under mineral oil.

### *In vitro* fertilization (IVF) of bovine

In this study, IVF was carried out as a control group. The IVF procedure was as described previously (Hosseini *et al*., 2016). Motile sperms were collected using swim down method with PureSperm® gradient. 1 × 10^6^/ml sperms were co-incubated with 10 matured COCs in 50 μl fertilization medium for 18 h at 38.5 °C, 5% CO_2_ and humidified atmosphere. After 18 h, presumptive zygotes were denuded of cumulus cells and cultured in BO-IVC medium (IVF Bioscience) at 38.5 °C, 5% CO_2_, 5% O_2_ and humidified air for 7–8 days under mineral oil. Derived blastocysts were used for Bisulfite sequencing PCR and gene expression analysis.

### SCNT of bovine

After denudation of matured oocytes with hyaluronidase and by vortexing, denuded oocytes were exposed to 5 mg/ml pronase for few seconds for removal of zona pellucida. The method of oocyte enucleation was done using manual oocyte enucleation using a fine pulled Pasteur pipette^36^. Briefly, zona free oocytes were incubated in TCM supplemented with 4 µg/ml demecolcine for 1 h in 38.5 °C. Then, cytoplasmic protrusion containing MII spindle, was removed by hand-held manual oocyte enucleation pipette. The procedures of nuclear replacement, electrofusion, oocyte activation were the same as described in preceding papers^36^. Subsequently, activated reconstructed oocytes were cultured inside well containing BO-IVC (IVF Bioscience) at 38.5 °C, 5% CO_2_, 5% O_2_ and humidified air for 7–8 days under mineral oil. To preclude aggregation of zona-free embryos, 20 µl droplets of BO-IVC were prepared under mineral oil in 3 cm Grainer® dishes. Six small wells were made by gently pressing a sterile steel rod with a round tip to the bottom of the culture dish and activated reconstructed oocytes were put in separate wells.

### Bisulfite sequencing PCR in IVF and SCNT derived blastocysts

Genomic DNA of blastocysts was extracted by salting-out method. All the detailed procedures of bisulfite sequencing PCR were exactly the same as mentioned for fibroblast cells in previous section.

### RNA extraction and Gene expression analysis

Total RNA from blastocysts was extracted by RNeasy Micro Kit (QIAGEN, cat.No. 74004) respectively according to manufacturer’s instruction. All the detailed procedures of RNA extraction and Gene expression analysis were exactly the same as mentioned for fibroblast cells in previous section.

### Statistical analysis

All experiments were repeated at least three times. Data is presented as mean ± S.E.M. Statistical significance was set at *P* < 0.05. One-way analyses of variance (ANOVA) was applied to study the effects of folate among various treatment groups (α = 0.05) followed by Tukey post-hoc test. The mRNA expression of MTHFR and BHMT genes in bovine fibroblast and kidney cells, and DNA methylation and mRNA expression of assessed genes in BFF-RPMI Folate+ and Folate− were analysed by independent samples t-test. GraphPad Prism (v.6.0.1) and IBM SPSS program (v.23, NY, USA) were used for statistical analysis.
